# Interface-Driven Carbon Fiber Reinforcement in Graphite Packing Rings for Enhanced Service Stability

**DOI:** 10.3390/ma19153226

**Published:** 2026-07-29

**Authors:** Yang Shi, Shihao Li, Xubo Bei, Cangeng Wang, Qi Liu, Daniu He, Leya Zhou, Yuting Huang, Peng Sun, Qiang Zhang, Shi He, Jun Jiang

**Affiliations:** 1 Zhejiang Zheneng Zhenhai Power Generation Co., Ltd., Ningbo 315221, China; shiyang2@zjenergy.com.cn (Y.S.); beixubo@zjenergy.com.cn (X.B.); wangcangeng@zjenergy.com.cn (C.W.); liuqi6@zjenergy.com.cn (Q.L.); hedaniu@zjenergy.com.cn (D.H.); zhouleya@zjenergy.com.cn (L.Z.); huangyut@zjenergy.com.cn (Y.H.); 2 Ningbo Institute of Materials Technology and Engineering, Chinese Academy of Sciences, Ningbo 315201, China; lishihao@nimte.ac.cn (S.L.); sunpeng417@nimte.ac.cn (P.S.); 3 University of Chinese Academy of Sciences, Beijing 100049, China

**Keywords:** flexible graphite packing rings, carbon fiber reinforcement, interface engineering, compressive and creep resistance, tribological stability

## Abstract

Flexible graphite packing rings are widely employed in high-temperature and high-pressure valve sealing systems owing to their intrinsic lubricity and thermal stability, yet their service reliability is often compromised by low mechanical strength, pronounced creep, and unstable tribological behavior under extreme conditions. Here, we present an interface-engineered strategy to enhance the service performance of graphite packing rings via reinforcement with surface-functionalized PAN-based carbon fibers (PAN-CFs; carbonized fibers derived from polyacrylonitrile precursors). Through controlled oxidative modification of carbon fibers combined with high-temperature graphite expansion, a three-dimensional reinforced graphite network with uniform fiber dispersion was constructed. The influence of PAN-based carbon fiber (PAN-CF) content (0–7 wt%) on compressive strength, thermal stability, friction behavior, and long-term durability was systematically evaluated. An optimal performance was achieved at 5 wt% PAN-CF, featuring a ~58% increase in compressive strength, a stable friction coefficient of 0.15–0.18, and enhanced creep resistance, while retaining over 78% of the initial strength after 1000 h of sustained loading. Microstructural and complementary structural analyses suggest that these improvements are associated with interfacial mechanical anchoring, fiber embedding, and load-transfer reinforcement enabled by fiber surface functionalization and the expanded graphite architecture. This work offers a practical material-level approach to improving the long-term reliability of graphite-based sealing components in demanding industrial environments.

## 1. Introduction

**Figure 1:**
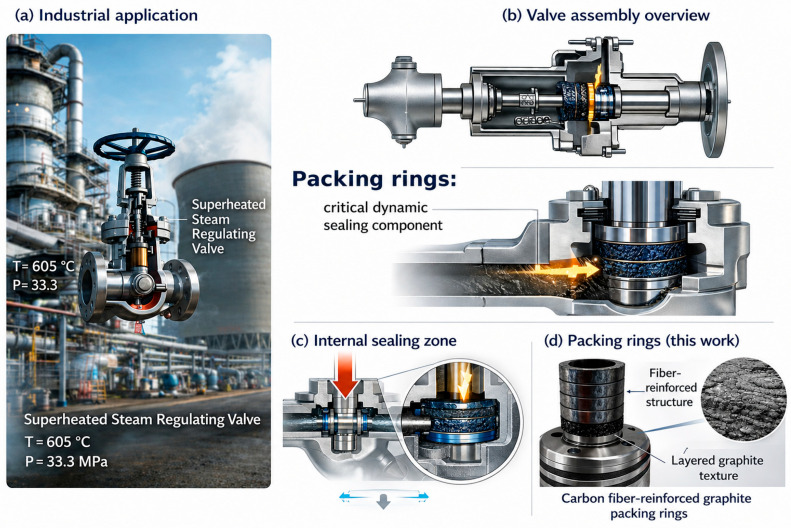
Application scenario and structural positioning of carbon fiber-reinforced graphite packing rings in high-temperature valve systems: (**a**) Industrial application of a superheated steam regulating valve operating under extreme conditions (*T* ≈ 605 °C, *p* ≈ 33.3 MPa). (**b**) Schematic overview of the valve assembly, highlighting the location of the packing rings as a critical dynamic sealing component. (**c**) Enlarged view of the internal sealing zone, illustrating the interaction between the valve stem motion and the packing rings under service conditions. (**d**) Carbon fiber-reinforced graphite packing rings developed in this work, featuring a layered graphite texture reinforced by a fiber network, as revealed by the representative microstructural inset.

High-temperature and high-pressure valves are indispensable components in energy, chemical, power-generation, and heavy machinery systems, where sealing reliability critically governs operational safety, service lifetime, and energy efficiency [1,2,3]. In supercritical and ultra-supercritical units, valves are routinely exposed to coupled conditions of elevated temperature, high pressure, and high flow velocity, imposing stringent demands on the wear resistance, load-bearing capability, and long-term stability of sealing components. In such systems, dynamic sealing at the valve stem is commonly achieved by packing materials housed within the stuffing box, among which packing rings serve as the primary sealing elements. These rings are required to maintain stable sealing performance, low friction, and adequate wear resistance under extreme conditions, including temperatures exceeding 300 °C, pressures above 30 MPa, and repeated sliding motion in corrosive environments. As illustrated in Figure 1, the packing rings are located around the valve stem and serve as critical dynamic sealing components under superheated steam conditions.

Flexible graphite packing rings have become the dominant sealing system for high-temperature valve applications owing to their excellent self-lubricity, chemical inertness, thermal stability, and elastic recovery [4,5,6]. Typically derived from natural flake graphite through intercalation and rapid thermal expansion, flexible graphite consists of worm-like expanded graphite structures that are subsequently consolidated by rolling or molding processes [7]. Sealing performance is mainly governed by interlayer van der Waals interactions and pressure-induced “labyrinth” and “bearing” effects. Despite these advantages, flexible graphite inherently suffers from low mechanical strength and poor resistance to creep and erosion. Under high compressive stress and prolonged service, the porous graphite framework is prone to plastic flow, structural collapse, and powdering, leading to preload relaxation, accelerated wear, and eventual sealing failure. These limitations become particularly pronounced under cyclic loading and frequent start–stop conditions.

To address valve sealing failure, previous studies have predominantly focused on structural optimization and alternative sealing concepts. Reviews and experimental studies have explored advanced sealing designs, including magnetic-fluid seals, gas-isolated sealing configurations, and optimized valve body–bonnet interfaces [8,9,10,11]. Structural refinements of packing geometries and gasket configurations have also demonstrated reduced leakage and improved load distribution [12,13]. While such approaches can alleviate localized sealing issues, they do not fundamentally resolve the intrinsic mechanical degradation and creep instability of graphite-based packing materials under extreme service conditions. Consequently, improving the material-level robustness of flexible graphite remains a critical challenge.

Incorporating reinforcing phases into graphite matrices offers a promising route to overcome these intrinsic limitations. Carbon fibers, in particular, possess high specific strength and modulus, excellent thermal stability, and good physicochemical compatibility with carbonaceous matrices, making them attractive reinforcement candidates [14,15,16]. Carbon fiber-reinforced composites have been widely adopted in aerospace and advanced engineering applications, often replacing traditional metallic materials [17,18,19,20]. Depending on processing routes and target applications, carbon fibers can be utilized in various forms, including unidirectional fibers, short fibers, and woven fabrics, and combined with diverse matrices to achieve tailored mechanical performance [21,22,23,24,25]. Through crack bridging, fiber pull-out, and efficient load transfer, carbon fibers can significantly enhance the compressive strength, fracture resistance, and wear performance of composite materials [26,27].

For the present packing-ring application, PAN-based carbon fibers were selected after considering both precursor-dependent fiber properties and practical engineering requirements. Carbon fibers are commonly classified according to precursor type, mainly including PAN-based, pitch-based, and cellulose/viscose-derived carbon fibers. PAN-based carbon fibers are currently the dominant commercial carbon fibers and are widely used because of their mature processing route, stable product quality, and balanced mechanical properties. Pitch-based carbon fibers, particularly mesophase pitch-based carbon fibers, are attractive for high-modulus and thermal-management applications, but their tensile strength is generally lower than that of high-performance PAN-based carbon fibers, and their processing cost may restrict large-scale use in sealing components. Cellulose- or viscose-derived carbon fibers may possess unique structural features, including corrugated morphologies, but their carbon yield is relatively low, and their industrial availability is more limited. In our preliminary comparison, PAN-CF-, MP-CF-, and PAN/MP-CF-reinforced expanded graphite composites were evaluated, and the PAN-CF system showed the best overall compressive, tribological, and thermal stability (Figure S1). Therefore, PAN-based carbon fibers were selected as the main reinforcement in this work.

Recent studies further demonstrate that interfacial engineering plays a decisive role in maximizing the reinforcing efficiency of carbon fibers. Surface functionalization, interfacial crystallization control, and multiscale hybrid reinforcement have been shown to substantially improve stress transfer and damage tolerance in polymer- and carbon-based composites [28,29,30]. In graphite-based systems, carbon nanofibers have been reported to markedly enhance compressive and flexural strength through interfacial bridging mechanisms [31]. However, for flexible graphite packing rings, direct mechanical blending of short carbon fibers often disrupts the anisotropic expanded graphite framework, resulting in poor fiber dispersion, increased porosity, and compromised sealing reliability.

Accordingly, developing a controllable and reproducible fabrication strategy that enables uniform carbon fiber dispersion while maintaining the advantageous expanded graphite framework, together with effective interfacial anchoring between fibers and graphite, is of critical importance. Achieving such a composite structure is essential for realizing graphite packing rings with enhanced compressive strength, reduced creep deformation, stable friction behavior, and long-term service reliability. In this work, we develop an interface-engineered carbon fiber-reinforced graphite packing ring by integrating surface-functionalized PAN-based carbon fibers into an expanded graphite framework. The novelty of this work lies in coupling PAN-CF surface functionalization with worm-like expanded graphite architecture design, thereby enabling fiber embedding, interfacial mechanical anchoring, and efficient load transfer while retaining the self-lubricating and thermally stable characteristics of expanded graphite. The purpose of this work is to clarify how PAN-CF surface treatment and PAN-CF content affect the microstructure, compressive behavior, thermal stability, tribological performance, and long-term service reliability of graphite packing rings for high-temperature and high-pressure sealing applications.

## 2. Experimental Section

**Figure 2:**
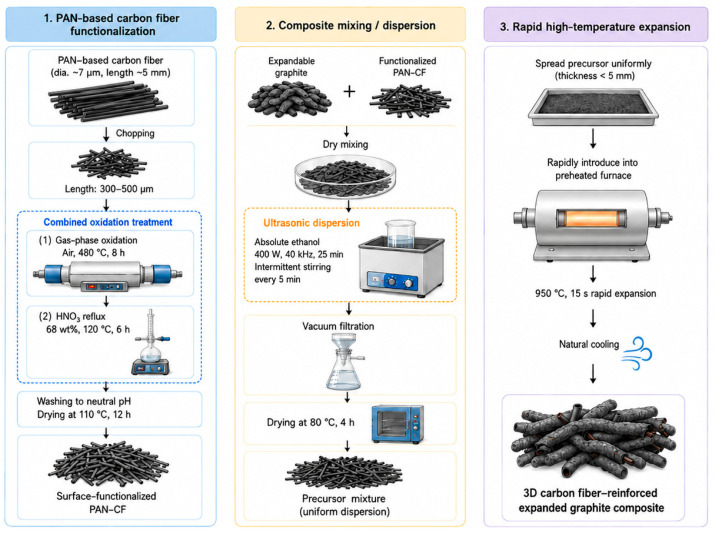
Schematic illustration of the fabrication process for interface-engineered carbon fiber–reinforced expanded graphite composites: (**1**) PAN-based carbon fibers were chopped to 300–500 μm and subjected to combined oxidation treatment, including gas-phase oxidation in air followed by HNO_3_ reflux. (**2**) The functionalized PAN-CFs were mixed with expandable graphite and dispersed in ethanol by ultrasonication, followed by vacuum filtration and drying to obtain a uniform precursor mixture. (**3**) The precursor mixture was rapidly expanded at 950 °C for 15 s to form a three-dimensional carbon fiber-reinforced expanded graphite framework.

The overall fabrication route for the interface-engineered PAN-CF/expanded graphite composites is schematically summarized in Figure 2. The process consists of PAN-CF surface functionalization, ultrasonic dispersion with expandable graphite, vacuum filtration and drying, rapid high-temperature expansion, and final hot-pressing into composite packing-ring specimens.

### 2.1. Surface Functionalization of PAN-Based Carbon Fibers

Commercial PAN-based carbon fibers (carbonized fibers derived from polyacrylonitrile precursors) with a purity of 97% were purchased from Shenzhen Jiasheng New Materials Co., Ltd., Shenzhen, China, and used as the reinforcing phase. The as-received fibers had an average filament diameter of approximately 7 μm and an initial length of approximately 5 mm. Prior to composite fabrication, the fibers were physically chopped to a length range of 300–500 μm. This length range was selected to balance reinforcement efficiency and dispersion uniformity: fibers that are too short may not effectively bridge graphite flakes or contribute to load transfer, whereas excessively long fibers tend to entangle during mixing, resulting in agglomeration and local structural defects. The selected chopped fibers therefore provide an appropriate aspect ratio for constructing a uniform fiber-reinforced expanded graphite framework. Basic information on the PAN-based carbon fibers is summarized in Table S1.

The surface oxidation was conducted via a combined gas-phase and liquid-phase treatment. First, the chopped carbon fibers were placed in a tubular furnace and heated to 480 °C at a rate of 5 °C·min^−1^ under flowing air, followed by isothermal holding for 8 h. After natural cooling to room temperature, the fibers were transferred into a three-neck flask containing 200 mL of 68 wt% nitric acid and refluxed at 120 °C for 6 h. The treated fibers were then thoroughly washed with deionized water until the filtrate reached neutral pH (≈7) and subsequently dried in a vacuum oven at 110 °C for 12 h.

### 2.2. Preparation of Expandable Graphite

High-purity natural flake graphite (≥99%) with particle sizes in the range of 150–270 μm was employed as the precursor. Expandable graphite was prepared via a chemical intercalation process. Specifically, graphite flakes were homogeneously mixed with potassium permanganate, followed by the gradual addition of concentrated nitric acid at 20 °C. The mass/volume ratio of graphite (g), nitric acid (mL), and KMnO_4_ (g) was maintained at 1.0:2.0:0.15.

After completion of the intercalation reaction, the product was repeatedly washed with deionized water until the pH of the filtrate reached approximately 5, filtered, and dried at 60 °C to obtain sulfur-free expandable graphite.

### 2.3. Mixing and Dispersion of Carbon Fibers and Expandable Graphite

Surface-functionalized PAN-CFs at the designed contents (0, 3, 5, and 7 wt%) were first dry-mixed with expandable graphite. The powder mixture was then dispersed in 200 mL of absolute ethanol in a 500 mL beaker and subjected to ultrasonication at a power of 400 W and a frequency of 40 kHz for 25 min. To prevent sedimentation, ultrasonication was intermittently paused every 5 min for manual stirring, ensuring homogeneous fiber dispersion within the graphite matrix.

### 2.4. High-Temperature Expansion and Formation of Composite Worm-like Structures

After dispersion, the suspension was vacuum-filtered and dried at 80 °C for 4 h to remove residual ethanol. The dried mixture was evenly spread in a quartz crucible with a thickness below 5 mm and rapidly introduced into a preheated muffle furnace at 950 °C. Thermal expansion was carried out for 15 s, during which the intercalated graphite underwent rapid expansion along the c-axis, forming worm-like expanded graphite structures interwoven with carbon fibers.

### 2.5. Hot-Pressing and Fabrication of Composite Packing Rings

The expanded graphite composite worms were loaded into a high-strength graphite mold and hot-pressed in air to obtain densified composite specimens and ring-like samples for characterization and performance testing. A preloading pressure of 5 MPa was first applied, followed by heating to 200 °C at a rate of 10 °C min^−1^. A final pressure of 12 MPa was then applied and maintained for 20 min to achieve densification. After cooling to below 60 °C, the molded composites were removed from the mold and machined into specimens or ring-like samples for structural characterization, compression testing, thermal stability evaluation, friction testing, and long-term service tests. High-speed braiding is a conventional industrial forming route for graphite packing products, but it was not used as a post-treatment step for the hot-pressed specimens evaluated in this work.

### 2.6. Characterization and Testing Methods

The microstructures of carbon fibers, graphite precursors, expanded graphite, and carbon fiber/expanded graphite composites were characterized using a field-emission scanning electron microscope (FEI Quanta FEG 250, FEI Company, Hillsboro, OR, USA). Powder or fractured composite specimens were mounted on conductive carbon tape before observation, and loose particles were gently removed to reduce charging and contamination. All SEM observations were conducted directly without sputter-coating with Au, Pt, or other conductive metals. SEM images were mainly collected using the secondary electron detector at an accelerating voltage of 5.0 kV.

The crystal structure was examined by X-ray diffraction (D8 Advance, Bruker AXS GmbH, Karlsruhe, Germany) using Cu Kα radiation (λ = 0.154 nm) over a 2θ range of 5–90°. Raman spectra were collected using a LabRAM HR Evolution Raman spectrometer with a 532 nm laser over the range of 1000–3000 cm^−1^. Fourier-transform infrared spectroscopy was performed using an iS 50 FTIR spectrometer (Thermo Fisher Scientific, Waltham, MA, USA) in attenuated total reflection (ATR) mode. Powder samples of graphite-related materials were placed directly on the ATR crystal, and spectra were recorded over the range of 4000–500 cm^−1^ to qualitatively examine the evolution of oxygen-containing functional groups during oxidation/intercalation and high-temperature expansion. Specific surface area and pore-structure characteristics were measured using an ASAP2460 analyzer after degassing the samples at 120 °C for 6 h.

Thermogravimetric analysis was performed using a TG 209 F1 thermogravimetric analyzer (NETZSCH-Gerätebau GmbH, Selb, Germany). Approximately 5–10 mg of each sample was placed in an alumina crucible and heated under a nitrogen atmosphere at a heating rate of 10 °C min^−1^. The mass retention was recorded as a function of temperature to evaluate the thermal stability of the composites.

Compression tests were conducted with reference to ASTM D695 [32] and GB/T 40398.1 [33] using a Zwick/Roell Z030 universal testing machine (ZwickRoell GmbH & Co. KG, Ulm, Germany) equipped with a 30 kN load cell. Because the specimens were graphite packing rings with non-standard annular geometries, axial compression was directly applied to the ring-like specimens at a constant displacement rate of 1 mm min^−1^. The load–displacement response was continuously recorded and converted into engineering compressive stress–strain curves. The compressive stress was calculated by dividing the applied axial force by the effective load-bearing annular area, A = π(*D*_o_^2^ − *D*_i_^2^)/4, where *D*_o_ and *D*_i_ are the outer and inner diameters of the ring specimen, respectively. The compressive strain was calculated as *ε* = Δ*h*/*h*_0_, where Δ*h* is the axial displacement and h_0_ is the initial specimen height. For each PAN-CF content, including 0, 3, 5, and 7 wt% PAN-CF, at least three independent composite packing-ring specimens were tested under the same conditions. The curves shown in this work are representative compression curves used to compare the deformation behavior and load-bearing capability of different compositions. The force-measurement accuracy of the testing system was Class 0.5 for the 30 kN load cell used, in accordance with DIN EN ISO 7500-1 [34] and ASTM E4 [35].

Tribological tests were conducted with reference to ASTM G99 [36] and GB/T 3960 [37] using a UMT-3 multifunctional high-temperature friction and wear tester (Center for Tribology Inc. (CETR), Campbell, CA, USA). The specimen mounting and contact configuration were adjusted according to the packing-ring geometry, while the basic principle of sliding-friction evaluation was retained. Friction tests were performed under a normal load of 10 N and a sliding velocity of 50 mm s^−1^. A polished stainless-steel counterface was used to simulate the contact surface of the valve stem. Each test was performed for 600 s at room temperature in ambient air, and the friction coefficient was continuously recorded as a function of sliding time. For the 1000 h long-term service tests, the composite packing-ring specimens were subjected to a sustained compressive stress of 10 MPa at room temperature in ambient air. Thermal aging was conducted at 250 °C for 1000 h in ambient air. Long-term friction stability was evaluated under the same contact configuration, normal load, sliding velocity, temperature, and atmosphere as the short-term friction tests unless otherwise specified. Additional details of the characterization and testing methods are summarized in Table S2.

### 2.7. Use of Generative AI for Schematic Figure Preparation

Figure 1 and Figure 2 were prepared with the assistance of a generative AI tool. The authors provided text-based prompts describing the application scenario of graphite packing rings in high-temperature valve systems and the fabrication workflow of the PAN-CF/expanded graphite composites. The AI-generated schematic outputs were then carefully checked, corrected, edited, and finalized by the authors to ensure scientific accuracy, consistency with the experimental procedure, and absence of spelling or grammatical errors. No copyrighted third-party images or published figures were used as input.

## 3. Results and Discussion

### 3.1. Surface Functionalization of Carbon Fibers and Its Interfacial Implications

Surface modification has been widely used to improve the interfacial interaction of carbon fibers in composite systems by tailoring surface morphology, surface activity, and interfacial load transfer [23,28,38,39]. In this work, the combined gas-phase and liquid-phase oxidation treatment was selected based on this general principle and our preliminary process optimization. The purpose of this treatment was not to change the bulk dimensions of the fibers but to modify defect-rich surface regions and produce roughened surface features for subsequent composite formation.

**Figure 3:**
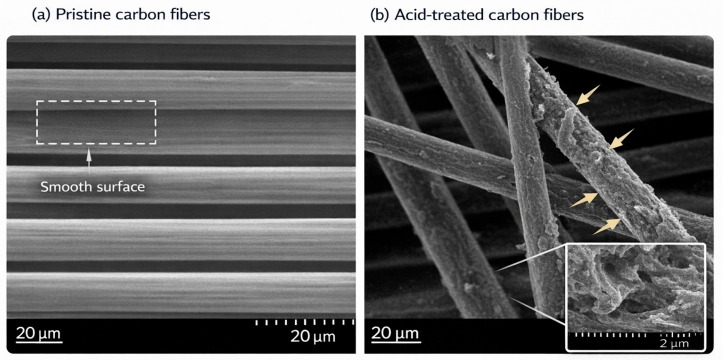
SEM images of PAN-based carbon fibers before and after acid oxidation treatment: (**a**) Pristine carbon fibers exhibit relatively smooth surfaces with well-defined longitudinal grooves. (**b**) Acid-treated carbon fibers show pronounced surface roughening with nanoscale grooves and etched features. The apparent local thickening after treatment is attributed to surface roughening and imaging effects rather than a confirmed increase in filament diameter.

Figure 3 compares the surface morphology of pristine and treated PAN-based carbon fibers. The pristine fibers exhibit relatively smooth surfaces with well-defined longitudinal grooves, which are typical of commercial PAN-based carbon fibers. After the combined oxidation treatment, the fiber surface becomes visibly rougher, with nanoscale grooves, pits, and etched features. The treated fibers may appear locally thicker in the SEM image; however, this apparent change should not be interpreted as a confirmed increase in filament diameter, but rather as a consequence of surface roughening, random fiber orientation, partial overlap, and imaging perspective.

The roughened morphology is consistent with selective etching of amorphous or defect-rich regions on the carbon fiber surface during acid treatment. Such surface features may increase the number of potential contact and anchoring sites between the fibers and expanded graphite flakes. Therefore, the discussion is limited to the directly observed morphology evolution from relatively smooth pristine fibers to roughened oxidized fibers with etched grooves and pits.

This surface roughening is expected to benefit mechanical interlocking at the fiber–graphite interface during composite formation. However, because direct interfacial shear or pull-out tests were not performed in this work, we have revised the wording to avoid overclaiming and only use the SEM observations as morphological evidence for improved interfacial anchoring potential.

### 3.2. High-Temperature Expansion Behavior of Graphite and Structural Implications

The structural characteristics of the graphite matrix play a critical role in determining whether effective fiber reinforcement can be realized. High-temperature expansion transforms the microstructure of graphite and creates a three-dimensional framework that can provide spatial accommodation and interfacial contact sites for fiber incorporation.

**Figure 4:**
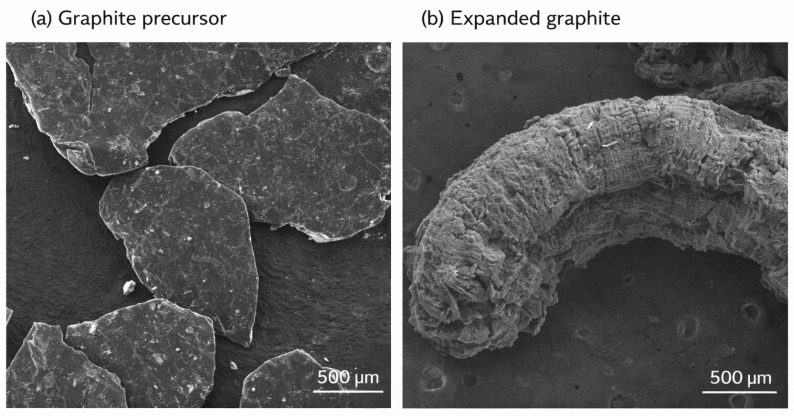
SEM images showing the morphological evolution of graphite before and after high-temperature expansion: (**a**) The graphite precursor exhibits a dense lamellar structure with closely stacked flakes and limited porosity. (**b**) After rapid thermal expansion, graphite transforms into a worm-like, three-dimensional porous architecture composed of loosely stacked and highly corrugated graphite flakes.

As shown in Figure 4a, the graphite precursor exhibits a dense lamellar morphology with closely stacked flakes. XRD analysis further reveals the corresponding interlayer structural evolution during oxidation/intercalation and high-temperature expansion (Figure S2). Pristine graphite shows a sharp (002) diffraction peak at 2θ ≈ 26.55°, corresponding to an interlayer spacing of approximately 0.34 nm according to Bragg’s law. After oxidative intercalation, the original sharp graphite (002) peak becomes markedly weakened and broadened, while an additional low-angle diffraction feature appears at approximately 2θ ≈ 13.37°, indicating enlarged interlayer spacing associated with oxidation/intercalation. After high-temperature expansion, the (002) peak reappears near 2θ ≈ 26.55° with reduced intensity and increased width, suggesting that the basic graphite layered structure is retained while the stacking order is partially disrupted during expansion.

After rapid high-temperature expansion, graphite undergoes a clear morphological transformation from compact lamellar stacking to a worm-like porous architecture. As shown in Figure 4b, the expanded graphite is composed of loosely stacked and highly corrugated graphite flakes, with visible interflake gaps and cavities distributed throughout the framework. Additional SEM images at different magnifications are provided in Figure S3, further confirming the morphological evolution from relatively compact graphite oxide to loose, expanded graphite. These SEM observations directly support the formation of a three-dimensional expanded graphite network.

BET analysis further quantifies the pore-structure evolution associated with high-temperature expansion (Figure S4). The specific surface area increases from approximately 2.58 m^2^ g^−1^ for graphite oxide to approximately 14.43 m^2^ g^−1^ for expanded graphite, confirming the development of a more open and porous framework after high-temperature expansion. This increase in accessible surface area and pore volume is consistent with the SEM-observed worm-like morphology and provides additional spatial accommodation for carbon fiber incorporation.

The expanded graphite framework exhibits a continuous worm-like morphology composed of interconnected and corrugated graphite flakes, providing a structural basis for fiber incorporation and interfacial contact during composite formation. XRD and Raman results further indicate that graphite-related structural features remain present after expansion, although the stacking order and defect state are modified during oxidation/intercalation and high-temperature treatment (Figures S2 and S5).

FTIR spectra were further used as qualitative supporting evidence for the evolution of oxygen-containing groups during graphite oxidation/intercalation and thermal expansion (Figure S6). The spectra were normalized and vertically shifted for comparative visualization, which is a commonly used presentation method for FTIR spectra of related carbonaceous samples and does not alter the peak positions or characteristic spectral features. For graphite oxide, the broad absorption band at approximately 3200–3600 cm^−1^ can be assigned to O–H stretching, while the bands near approximately 1700–1730 cm^−1^ and 1000–1250 cm^−1^ are related to C=O and C–O/C–O–C vibrations, respectively. These features indicate the introduction of oxygen-containing groups during oxidative intercalation. After high-temperature expansion, the intensities of these oxygen-related bands decrease markedly, especially for samples expanded at higher temperatures, suggesting partial thermal decomposition and removal of oxygen-containing groups. Therefore, the FTIR results are used only as qualitative evidence for surface-chemical evolution, while the main structural interpretation is supported by SEM, XRD, BET, and Raman analyses.

### 3.3. Microstructural Characteristics of Carbon Fiber/Expanded Graphite Composites

Following the formation of a three-dimensional expanded graphite framework, the distribution and interfacial characteristics of surface-functionalized carbon fibers within this matrix were examined by SEM. The SEM results provide direct morphological evidence of the multiscale composite architecture formed by integrating acid-treated carbon fibers with expanded graphite worms.

**Figure 5:**
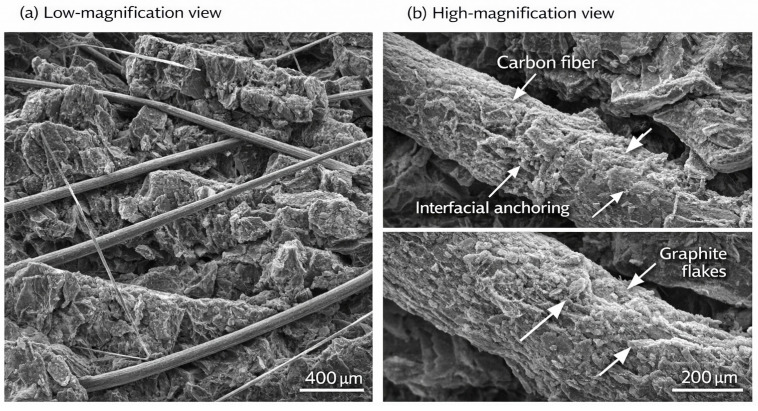
SEM images illustrating the microstructure of the composite composed of surface-treated PAN-based carbon fibers and expanded graphite: (**a**) Low-magnification image showing the overall carbon fiber-reinforced expanded graphite architecture. (**b**) Higher-magnification images showing carbon fiber embedding and interfacial contact within the expanded graphite framework. Scale bars are shown in the figure.

As displayed in Figure 5, at low magnification, expanded graphite forms a continuous three-dimensional skeleton composed of interwoven worm-like structures. Acid-treated short carbon fibers are embedded within this framework without obvious large-scale agglomeration, indicating that the adopted dispersion and expansion process can promote fiber incorporation into the graphite matrix.

Higher-magnification observations reveal direct contact between the roughened carbon fiber surfaces and expanded graphite flakes. The nanoscale grooves and etched features on the treated fibers may provide additional anchoring sites, while the corrugated graphite flakes create local contact regions around the fibers. These observations support the possibility of mechanical interlocking, but they are not treated as direct quantitative proof of enhanced interfacial adhesion.

The improved composite integrity should therefore be understood as the result of combined morphological, structural, and surface-chemical factors. SEM provides direct morphology evidence, whereas XRD, BET, Raman, and FTIR results in Figures S2 and S4–S6 support the graphite structural evolution, pore development, defect evolution, and surface functional-group changes. These results collectively suggest favorable conditions for interfacial contact and mechanical anchoring, although direct interfacial adhesion testing was not performed in this work.

### 3.4. Effect of PAN-Based Carbon Fiber Content on Compressive, Thermal, and Tribological Properties

Following the establishment of a hierarchical composite architecture composed of expanded graphite and surface-functionalized carbon fibers, the influence of PAN-CF content on the macroscopic performance of the composites was systematically investigated. The results demonstrate that PAN-CF content plays a decisive role in governing compressive behavior, thermal stability, and tribological performance.

**Figure 6:**
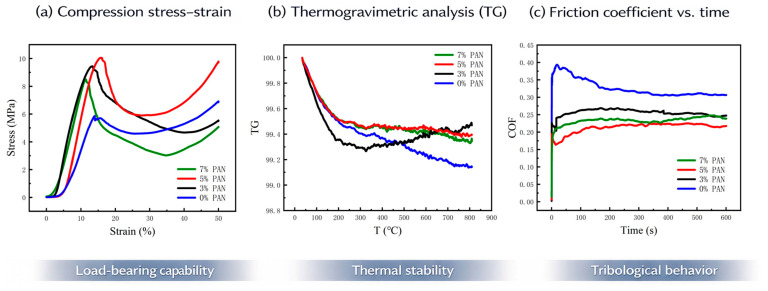
Effect of PAN-CF content on the compressive, thermal, and tribological properties of carbon fiber-reinforced expanded graphite composites: (**a**) Compressive stress–strain curves showing the evolution of load-bearing capability with increasing PAN-CF content. (**b**) Thermogravimetric (TG) curves recorded under a nitrogen atmosphere, illustrating the influence of PAN-CF content on the thermal stability of the composites. (**c**) Friction coefficient as a function of sliding time, revealing the effect of PAN-CF content on friction reduction and tribological stability.

Compressive stress–strain curves reveal a pronounced composition-dependent evolution of mechanical properties. For each PAN-CF content, including 0, 3, 5, and 7 wt% PAN-CF, at least three independently prepared composite packing-ring specimens were tested under the same conditions. The curves shown in Figure 6a are representative compression curves selected to compare the deformation behavior and load-bearing response of different PAN-CF contents. To provide a quantitative comparison, the extracted compressive strength values are reported as mean ± standard deviation. The compressive strength of the PAN-CF-free sample was 6.3 ± 0.4 MPa, whereas that of the 5 wt% PAN-CF composite increased to 10.0 ± 0.5 MPa, corresponding to an improvement of approximately 58%. The 3 and 7 wt% PAN-CF composites exhibited compressive strengths of 8.1 ± 0.5 MPa and 7.4 ± 0.6 MPa, respectively. These statistical results confirm that the 5 wt% PAN-CF composite possesses the highest load-bearing capability among the tested compositions, while excessive PAN-CF addition slightly reduces the strengthening efficiency, probably owing to local fiber aggregation or structural discontinuities.

Thermogravimetric analysis shows that all composites exhibit excellent thermal stability under nitrogen, with only limited mass loss over the tested temperature range. Approximately 5–10 mg of each sample was used for TG analysis. It should be noted that the mass-retention differences among the composites are relatively small; therefore, the TG results are mainly used to demonstrate the excellent overall thermal stability of the carbonaceous composites, rather than to claim a large thermal-stability enhancement. The 5 wt% PAN-CF composite shows slightly higher mass retention than the PAN-CF-free sample, suggesting a modest improvement in thermal stability.

Tribological measurements further highlight the beneficial role of PAN-CF reinforcement in regulating friction behavior. The steady-state friction coefficient was calculated from the stable sliding region of the friction curves and is reported as mean ± standard deviation. The PAN-CF-free composite shows a relatively high and unstable friction coefficient of 0.42 ± 0.03, characteristic of pronounced stick–slip behavior. With increasing PAN-CF content, the steady-state friction coefficient decreases. The composite containing 5 wt% PAN-CF achieves a much lower and more stable value of 0.17 ± 0.01, corresponding to an approximately 60% reduction compared with the PAN-CF-free composite. The 3 and 7 wt% PAN-CF composites show steady-state friction coefficients of 0.23 ± 0.02 and 0.21 ± 0.02, respectively. This improvement is mainly associated with the formation of a stable carbonaceous transfer layer during sliding and the enhanced load-bearing capacity of the composite, which helps preserve surface integrity under sustained friction. The statistical comparison supports the conclusion that the 5 wt% PAN-CF sample provides the most stable friction behavior among the tested compositions.

### 3.5. Long-Term Service Performance Under 1000 H Testing

**Figure 7:**
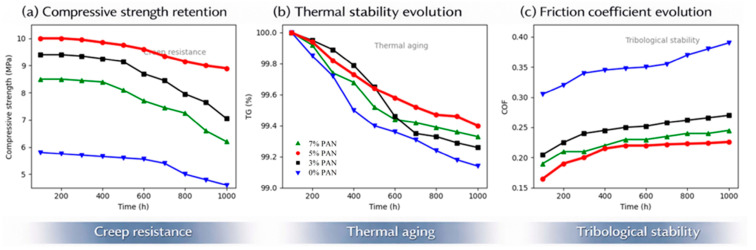
Long-term service performance of carbon fiber-reinforced expanded graphite composites with different PAN-CF contents: (**a**) Evolution of compressive strength during 1000 h of sustained loading. (**b**) Mass retention during long-term thermal aging. (**c**) Friction coefficient evolution during long-term sliding tests. The curves corresponding to 0, 3, 5, and 7 wt% PAN-CF are indicated by the legends. The 5 wt% PAN-CF composite exhibits the best overall long-term stability.

As shown in Figure 7, the long-term service performance of the composites strongly depends on the PAN-CF content. The different curves in Figure 7 correspond to 0, 3, 5, and 7 wt% PAN-CF, respectively, as indicated by the legends. Figure 7a compares the evolution of compressive strength during 1000 h of sustained loading, Figure 7b shows the mass retention during long-term thermal aging, and Figure 7c presents the friction coefficient evolution during long-term sliding tests. Among all compositions, the 5 wt% PAN-CF composite exhibits the best overall stability, combining high strength retention, excellent thermal stability, and a low, stable friction coefficient.

Under sustained loading, all composites exhibit stress relaxation to some extent; however, the magnitude of degradation decreases markedly with increasing PAN-CF content. After 1000 h of sustained loading, the PAN-CF-free sample retained only 42.3 ± 3.1% of its initial compressive strength, whereas the 5 wt% PAN-CF composite retained 78.1 ± 3.4%. The corresponding strength-retention values of the 3 and 7 wt% PAN-CF composites were 68.4 ± 3.6% and 59.7 ± 4.2%, respectively. All samples undergo a relatively rapid degradation stage during the initial 0–200 h, corresponding to interfacial rearrangement and microdefect evolution. Beyond this period (200–1000 h), the degradation rate of PAN-CF-containing composites decreases substantially, suggesting the establishment of a more stable internal stress equilibrium. The enhanced long-term mechanical stability is attributed to the carbon fiber network within the composite, which helps redistribute stress concentrations and suppress interfacial sliding within the expanded graphite framework.

Long-term thermal aging tests reveal distinct differences in thermal stability evolution among composites with varying PAN-CF contents. Although all samples experience slight mass loss after 1000 h of thermal exposure, increasing PAN-CF content retards degradation to some extent. The PAN-CF-free sample shows a mass retention of 99.12 ± 0.05%, whereas the 5 wt% PAN-CF composite shows the highest mass retention of 99.68 ± 0.04%. The 3 and 7 wt% PAN-CF composites exhibit mass retentions of 99.54 ± 0.06% and 99.36 ± 0.07%, respectively. Because the differences in mass retention are relatively small, the thermal-aging result is interpreted as a modest improvement rather than a dominant strengthening mechanism. The thermal degradation process shows a two-stage behavior: an initial rapid mass-loss stage (0–200 h), followed by a slower degradation stage (200–1000 h). In the latter stage, PAN-CF-containing composites display reduced mass-loss rates. The improvement is attributed to the thermally stable carbonized fiber network and strengthened expanded graphite framework, rather than to any PAN precursor-related molecular reactions.

The long-term evolution of friction behavior further highlights the stabilizing effect of PAN-CF reinforcement. The PAN-CF-free composite shows rapid friction deterioration during the initial stage, with the friction coefficient increasing from approximately 0.20 to a final value of 0.38 ± 0.03 and remaining highly unstable thereafter. In contrast, the composite containing 5 wt% PAN-CF maintains a low friction coefficient of 0.17 ± 0.01 after 1000 h of testing, with fluctuations below ±0.02 during the test. The final friction coefficients of the 3 and 7 wt% PAN-CF composites are 0.24 ± 0.02 and 0.27 ± 0.02, respectively. These statistical data further support the conclusion that the 5 wt% PAN-CF composite exhibits the best overall long-term tribological stability. This enhanced stability arises from the sustained integrity of a self-lubricating carbonaceous transfer film at the sliding interface and the improved structural support provided by the carbonized PAN-CF/expanded graphite framework. The mechanism is therefore attributed to carbon fiber reinforcement, interfacial mechanical anchoring, and suppression of graphite flake slippage, rather than to polymer-chain mobility or other PAN precursor-related behavior.

Overall, the above results establish a clear relationship between interfacial structure and service performance. Surface oxidation transforms the PAN-based carbon fibers from relatively smooth filaments into roughened fibers with etched grooves and anchoring sites, while high-temperature expansion converts compact graphite flakes into a worm-like porous framework. The combination of these two structural features enables the treated carbon fibers to be more effectively embedded and mechanically anchored within the expanded graphite network. As a result, the composite packing rings exhibit improved load transfer, suppressed graphite flake slippage, and a more stable frictional interface. These microstructural advantages account for the simultaneous enhancement in compressive strength, thermal stability, friction stability, and long-term durability. Therefore, the novelty of this work lies not in the simple addition of carbon fibers but in constructing an interface-engineered PAN-CF/expanded graphite architecture specifically designed for high-temperature and high-pressure dynamic sealing applications.

## 4. Conclusions

In summary, a robust and scalable strategy was developed to enhance the comprehensive service performance of flexible graphite packing rings through interface-engineered PAN-based carbon fiber reinforcement. Surface oxidation generated roughened fiber surfaces with etched grooves and pits, while high-temperature expansion produced a hierarchical expanded graphite framework capable of accommodating and anchoring short carbon fibers. The optimized composite containing 5 wt% PAN-CF exhibited enhanced compressive strength, stable low-friction behavior, improved thermal stability, and superior long-term service performance compared with the PAN-CF-free graphite matrix. These improvements are attributed to the combined effects of fiber bridging, mechanical interlocking, load transfer, and the stabilization of the carbonaceous transfer layer during sliding. The present work demonstrates the feasibility of using PAN-based carbon fibers to improve graphite packing rings for high-temperature and high-pressure valve sealing applications. Meanwhile, pitch-based and cellulose-derived carbon fibers, especially corrugated cellulose-derived carbon fibers, may provide alternative reinforcement routes and will be considered in future precursor-dependent comparative studies.

## Data Availability

The original contributions presented in this study are included in the article/Supplementary Material. Further inquiries can be directed to the corresponding authors.

## Supplementary Materials

The following supporting information can be downloaded at: https://www.mdpi.com/article/10.3390/ma19153226/s1, Figure S1: Comparison of expanded graphite composite packing rings reinforced by different carbon fibers. (a) Com-pressive stress–strain curves and (b) compressive stress at 15% deformation for PAN-CF, MP-CF, and PAN/MP-CF reinforced composites. (c) Friction coefficient evolution during 600 s dry sliding and (d) friction coefficient at 600 s. (e) TG curves from room temperature to 800 °C and (f) mass retention at 800 °C. The PAN-CF reinforced composite exhibits the best overall compressive, tribological, and thermal stability, supporting the selection of PAN-based carbon fibers in this work; Figure S2: XRD patterns of pristine graphite, graphite oxide (GO), and expanded graphite (EG). Pristine graphite exhibits a sharp (002) diffraction peak at 2θ ≈ 26.55°, corresponding to an interlayer spacing of approximately 0.34 nm according to Bragg’s law. After oxidative intercalation, the original sharp graphite (002) peak becomes markedly weakened and broadened, while an additional low-angle diffraction feature appears at approximately 2θ ≈ 13.37°, indicating enlarged interlayer spacing associated with oxidation/intercalation. After high-temperature expansion, the (002) peak reappears near 2θ ≈ 26.55° with reduced intensity and increased width, suggesting partial recovery of the graphite layered structure with decreased stacking order; Figure S3: SEM images of graphite oxide and expanded graphite at different magnifications. Graphite oxide shows compact flake-like morphology, whereas expanded graphite exhibits a loose worm-like framework with corrugated flakes, interflake gaps, and visible cavities; Figure S4: BET pore-size distribution and specific surface area of graphite oxide and expanded graphite. The specific surface area increases from approximately 2.58 m^2^ g^−1^ for graphite oxide to approximately 14.43 m^2^ g^−1^ for expanded graphite, confirming the development of a more porous structure after high-temperature expansion; Figure S5: Raman spectra of graphite-related samples. (a) Raman spectra of graphite, graphite oxide (GO), and expanded graphite (EG). (b) Raman spectra of expanded graphite prepared at different expansion temperatures. The D, G, and 2D bands reflect the defect structure and graphitic ordering of the samples during oxidation and high-temperature expansion; Figure S6: Normalized FTIR spectra of graphite-related samples before and after oxidation/intercalation and high-temperature ex-pansion. (a) FTIR spectra of graphite, graphite oxide (GO), and expanded graphite (EG). (b) FTIR spectra of expanded graphite prepared at different expansion temperatures. The spectra were replotted directly from the raw FTIR data without smoothing or curve fitting, and were normalized and vertically shifted only for comparative visualization. The oxygen-containing functional-group bands, including O–H, C=O, and C–O/C–O–C-related vibrations, are more pronounced in graphite oxide and become weakened after high-temperature expansion, consistent with thermal removal of oxygen-containing groups. The spectra are used as qualitative supporting evidence for surface-chemical evolution; Table S1: Basic information of the PAN-based carbon fibers used in this work; Table S2: Characterization and testing methods used in this work.
