# Supplementary material for: Interface-Driven Carbon Fiber Reinforcement in Graphite Packing Rings for Enhanced Service Stability
Source: Materials (Basel). 2026 Jul 29;19(15):3226. doi: 10.3390/ma19153226 (PMC13466271; doi:10.3390/ma19153226)
Supplement: Supplementary file 1 [file materials-19-03226-s001.zip › materials-4296808-supplementary.pdf]

materials

MDPI

Interface-Driven Carbon Fiber Reinforcement in Graphite Packing Rings for Enhanced Service Stability

Yang Shi [1,†], Shihao Li [2,†], Xubo Bei [1], Cangeng Wang [1], Qi Liu [1], Daniu He [1], Leya Zhou [1], Yuting Huang [1], Peng Sun [2], Qiang Zhang [2,3,*], Shi He [1,*] and Jun Jiang [2,3,*]

[1]Zhejiang Zheneng Zhenhai Power Generation Co., Ltd. Ningbo 315221, China.
[2]Ningbo Institute of Materials Technology and Engineering, Chinese Academy of Sciences, Ningbo 315201, China.
[3]University of Chinese Academy of Sciences, Beijing 100049, China.
[†]These authors contributed equally to this work.
* Correspondence: authors. Email: qiangzhang@nimte.ac.cn; nb_heshi@163.com; jjun@nimte.ac.cn.

Supplementary Information

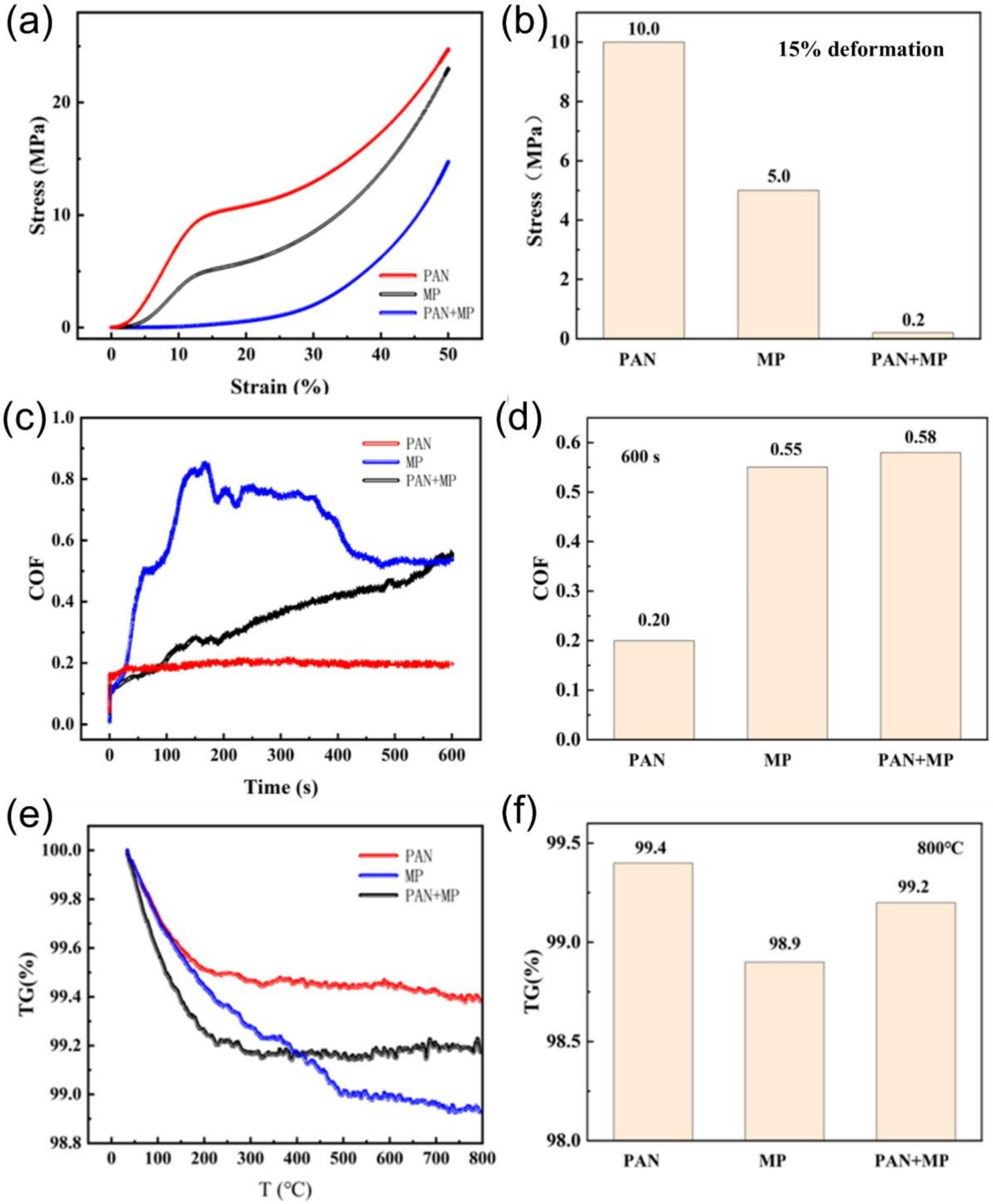

Figure S1. Comparison of expanded graphite composite packing rings reinforced by different carbon fibers. (a) Compressive stress–strain curves and (b) compressive stress at 15% deformation for PAN-CF, MP-CF, and PAN/MP-CF reinforced composites. (c) Friction coefficient evolution during 600 s dry sliding and (d) friction coefficient at 600 s. (e) TG curves from room temperature to 800 °C and (f) mass retention at 800 °C. The PAN-CF reinforced composite exhibits the best overall compressive, tribological, and thermal stability, supporting the selection of PAN-based carbon fibers in this work.

Table S1. Basic information of the PAN-based carbon fibers used in this work.

Parameter	Value / description	Source
Fiber type	PAN-based carbon fiber	This work
Supplier	Shenzhen Jiasheng New Materials Co., Ltd.	Experimental material record
Purity	97%	Supplier information
Initial length	~5 mm	This work
Chopped length	300–500 μm	This work
Filament diameter	~7 μm	SEM observation in this work; consistent with the reported typical range of 5–7 μm
Initial morphology	Smooth surface with longitudinal grooves	SEM observation in this work
Morphology after oxidation	Roughened surface with nanoscale grooves and etched features	SEM observation in this work
Tensile strength	Typically 1.0–10.0 GPa, preferably 1.5–7.0 GPa	Reported range for PAN-based carbon fibers in related patent/literature; not measured for this batch
Tensile modulus	Not provided / not measured	This work
Density	Not provided / not measured	This work

Note: The tensile strength range is a typical range reported for PAN-based carbon fibers in patent/literature sources and does not represent the measured or supplier-certified value of the specific batch used in this work.

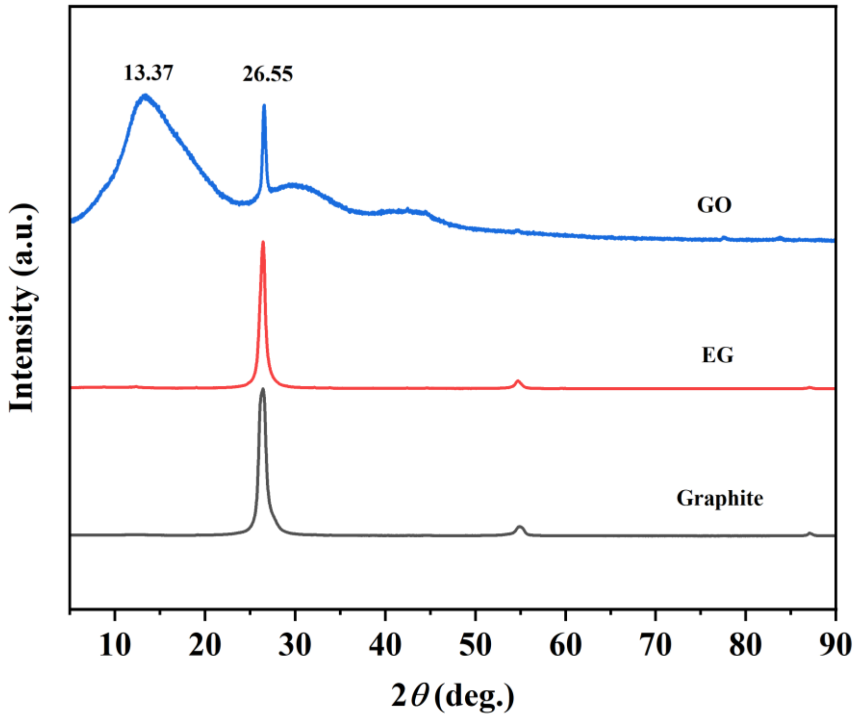

Figure S2. XRD patterns of pristine graphite, graphite oxide (GO), and expanded graphite (EG). Pristine graphite exhibits a sharp (002) diffraction peak at 2θ ≈ 26.55°, corresponding to an interlayer spacing of approximately 0.34 nm according to Bragg's law. After oxidative intercalation, the original sharp graphite (002) peak becomes markedly weakened and broadened, while an additional low-angle diffraction feature appears at approximately 2θ ≈ 13.37°, indicating enlarged interlayer spacing associated with oxidation/intercalation. After high-temperature expansion, the (002) peak reappears near 2θ ≈ 26.55° with reduced intensity and increased width, suggesting partial recovery of the graphite layered structure with decreased stacking order.

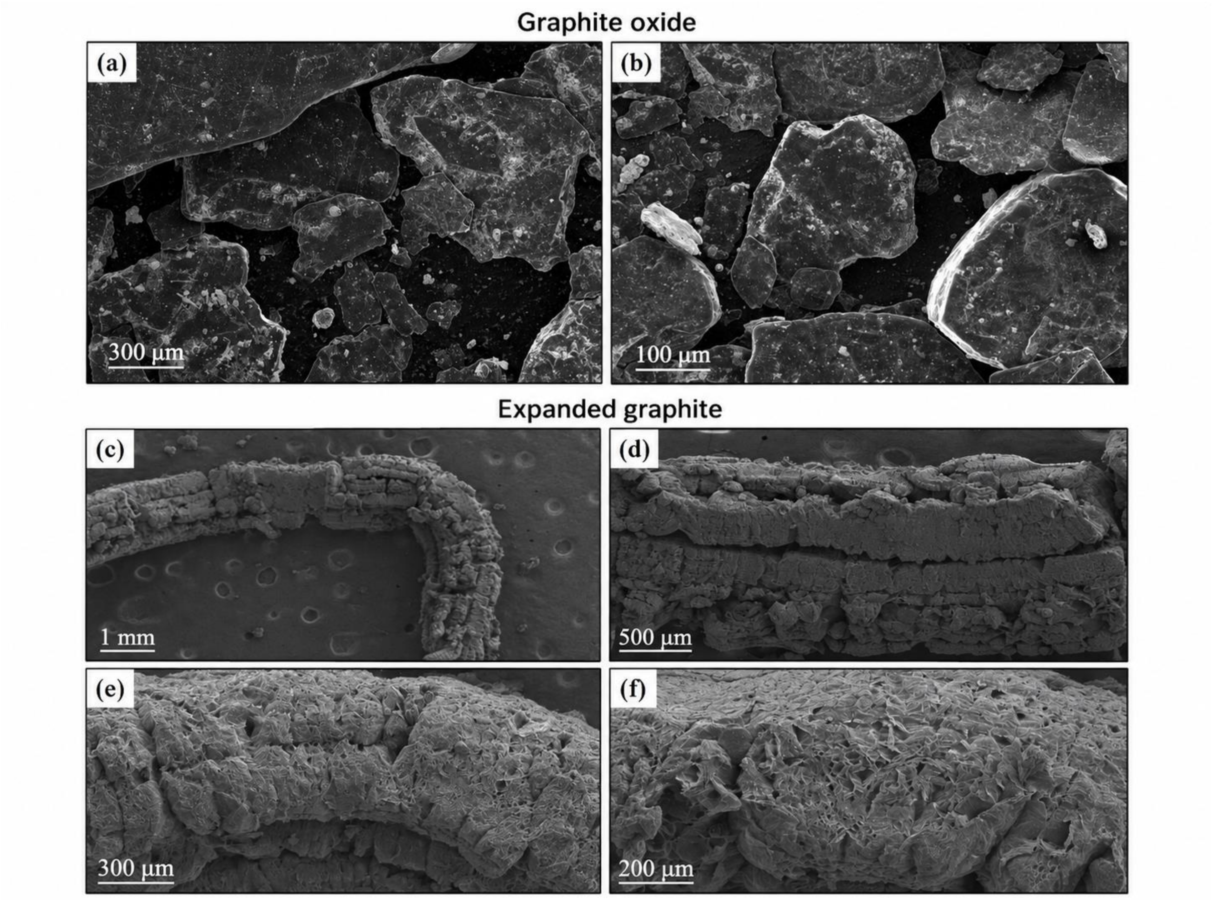

Figure S3. SEM images of graphite oxide and expanded graphite at different magnifications. Graphite oxide shows compact flake-like morphology, whereas expanded graphite exhibits a loose worm-like framework with corrugated flakes, interflake gaps, and visible cavities.

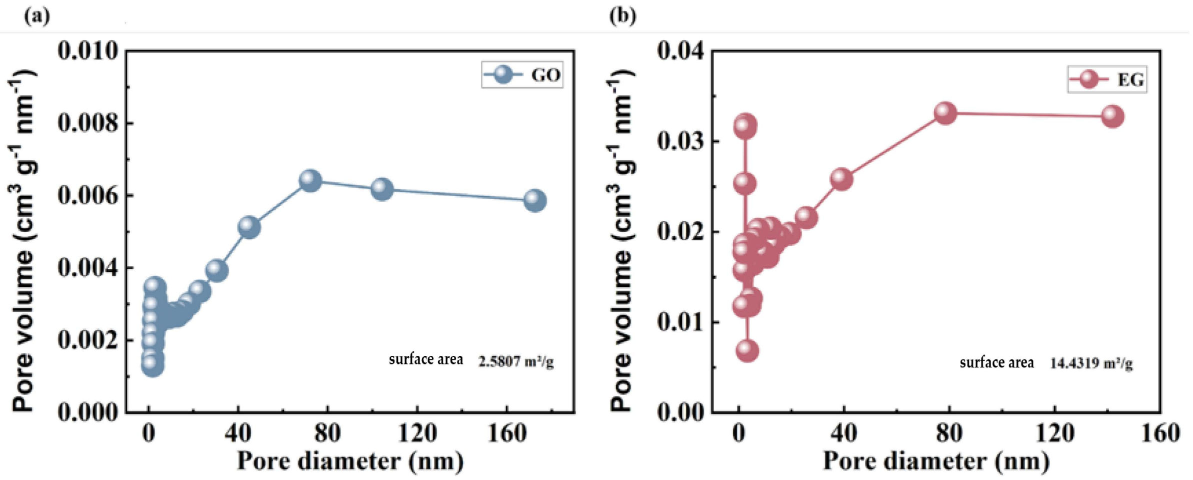

Figure S4. BET pore-size distribution and specific surface area of graphite oxide and expanded graphite. The specific surface area increases from approximately 2.58 m² g⁻¹ for graphite oxide to approximately 14.43 m² g⁻¹ for expanded graphite, confirming the development of a more porous structure after high-temperature expansion.

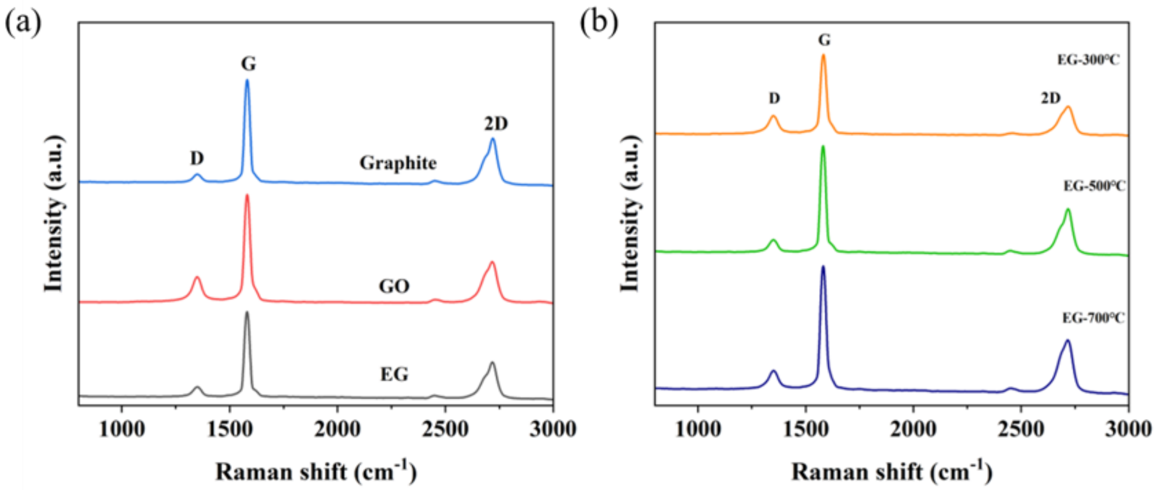

Figure S5. Raman spectra of graphite-related samples. (a) Raman spectra of graphite, graphite oxide (GO), and expanded graphite (EG). (b) Raman spectra of expanded graphite prepared at different expansion temperatures. The D, G, and 2D bands reflect the defect structure and graphitic ordering of the samples during oxidation and high-temperature expansion.

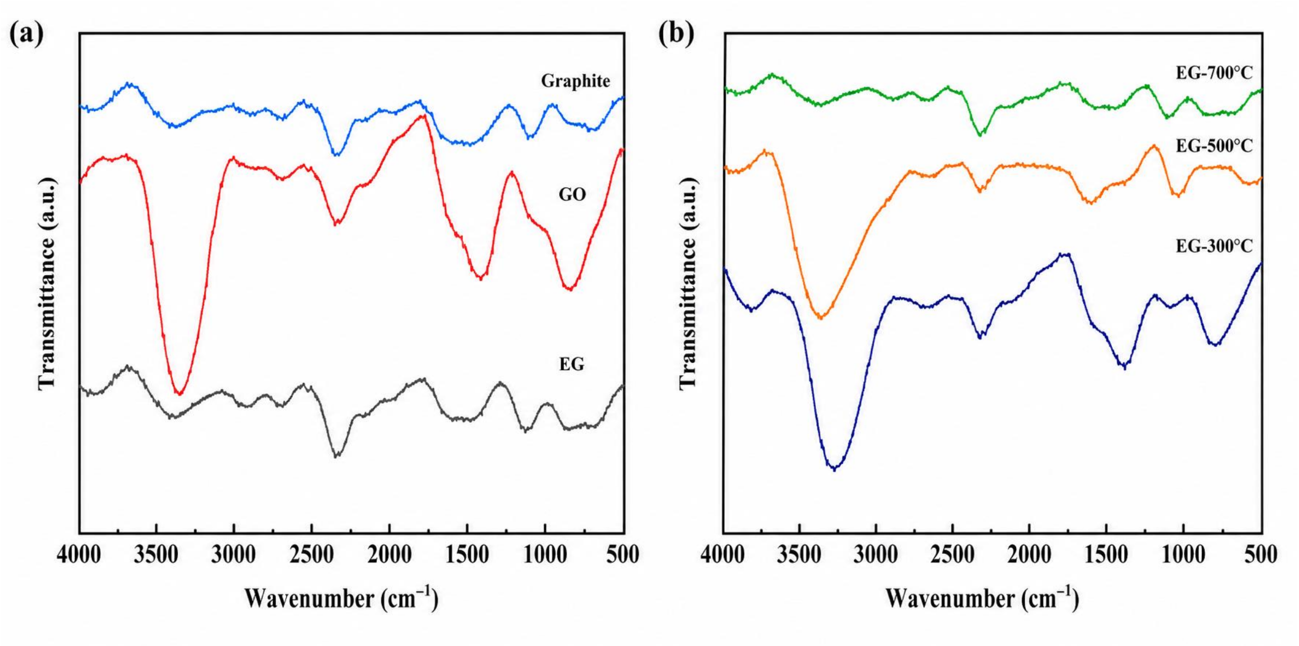

Figure S6. Normalized FTIR spectra of graphite-related samples before and after oxidation/intercalation and high-temperature expansion. (a) FTIR spectra of graphite, graphite oxide (GO), and expanded graphite (EG). (b) FTIR spectra of expanded graphite prepared at different expansion temperatures. The spectra were replotted directly from the raw FTIR data without smoothing or curve fitting, and were normalized and vertically shifted only for comparative visualization. The oxygen-containing functional-group bands, including O–H, C=O, and C–O/C–O–C-related vibrations, are more pronounced in graphite oxide and become weakened after high-temperature expansion, consistent with thermal removal of oxygen-containing groups. The spectra are used as qualitative supporting evidence for surface-chemical evolution.

Table S2. Characterization and testing methods used in this work.

Method	Sample / preparation	Instrument	Key parameters	Purpose
SEM	Carbon fibers, graphite precursors, expanded graphite, and fractured composite specimens mounted on conductive carbon tape. Loose particles were gently removed before observation. No Au, Pt, or other metal coating was applied.	FEI Quanta FEG 250, FEI Company, USA	Secondary electron imaging; accelerating voltage mainly 5.0 kV; direct observation without sputter coating.	Observe fiber surface morphology, graphite expansion morphology, fiber dispersion, and fiber/graphite interfacial contact
XRD	Powder samples of graphite-related materials flattened on the sample holder.	D8 Advance, Bruker, Germany	Cu Kα radiation, λ = 0.154 nm; 2θ range: 5–90°	Identify crystal structure and evaluate graphite interlayer structure
Raman spectroscopy	Graphite-related powder samples placed on glass slides.	LabRAM HR Evolution, Horiba, Japan	Laser wavelength: 532 nm; wavenumber range: 1000–3000 cm−1	Evaluate defect density and graphitic ordering through D, G, and 2D bands
FTIR spectroscopy	Graphite-related powder samples placed directly on the ATR crystal.	iS 50, Thermo Fisher Scientific, USA	ATR mode; spectral range: 4000–500 cm^{-1}. Spectra were replotted from raw data without smoothing or curve fitting, then normalized and vertically shifted for comparative visualization.	Qualitatively examine oxygen-containing functional groups introduced during oxidation/intercalation and weakened or removed during high-temperature expansion.
BET analysis	Samples degassed at 120 °C for 6 h before measurement.	ASAP2460, Micromeritics, USA	BET and t-plot methods	Determine specific surface area and pore-structure characteristics
TG analysis	Approximately 5–10 mg of sample placed in an alumina crucible.	TG 209 F1, NETZSCH-Gerätebau GmbH, Germany	Heating rate: 10 °C min−1; nitrogen atmosphere	Evaluate thermal stability and mass retention of the composites
Compression test	Composite packing-ring specimens tested under axial	Zwick/Roell Z030, Zwick Roell, Germany	With reference to ASTM D695 and GB/T 40398.1; Zwick/Roell Z030	Evaluate compressive behavior, load-bearing capability,

	compression. At least three independent specimens were tested for each PAN-CF content.		equipped with a 30 kN load cell; displacement rate: 1 mm min^{-1}; representative stress–strain curves. Engineering stress calculated as $\sigma = F/A$, where $A = \pi(D_o^2 - D_i^2)/4$; engineering strain calculated as $\varepsilon = \Delta h/h_0$. Force-measurement accuracy: Class 0.5 for the load cell used, in accordance with DIN EN ISO 7500-1 and ASTM E4.	resistance to plastic deformation, and long-term strength retention.
Friction test	Composite packing-ring specimens tested under sliding contact.	UMT-3 multi-functional high-temperature friction and wear tester, USA	With reference to ASTM G99 and GB/T 3960; normal load: 10 N; sliding velocity: 50 mm s^{-1}; duration: 600 s; polished stainless-steel counterface; room temperature; ambient air.	Evaluate tribological behavior, friction coefficient evolution, transfer-layer stability, and friction stability.
Long-term service test	Composite specimens with different PAN-CF contents tested for long-term stability.	Zwick/Roell Z030, TG 209 F1, and UMT-3	Testing duration: 1000 h. Sustained compression: 10 MPa at room temperature in ambient air. Thermal aging: 250 °C in ambient air. Long-term friction stability: same contact configuration, normal load, sliding velocity, temperature, and atmosphere as the short-term friction test unless otherwise specified.	Evaluate long-term compressive strength retention, thermal aging behavior, and friction coefficient evolution under service-simulation conditions.
